# Two synchronous malignant tumors of the pancreas: a case report

**DOI:** 10.1186/s13256-017-1244-0

**Published:** 2017-03-28

**Authors:** W. S. L. De Silva, A. A. Pathirana, I. Prematilleke, S. A. P. D. Rajapakse, P. S. H. Hettiarachchi, D. S. Manawasinghe, B. K. Dassanayake

**Affiliations:** 1 Post Graduate Institute of Medicine, University of Colombo, Colombo, Sri Lanka; 2 Department of Surgery, Faculty of Medical Sciences, University of Sri Jayewardenepura, Nugegoda, Colombo Sri Lanka; 3 Department of Pathology, Faculty of Medical Sciences, University of Sri Jayewardenepura, Nugegoda, Colombo Sri Lanka; 4 Department of Radiology, Asiri Surgical Hospital, Colombo, Sri Lanka

**Keywords:** Synchronous primary pancreatic carcinoma, Total pancreaticoduodenectomy, Ampullary carcinoma

## Abstract

**Background:**

Only a limited number of multiple synchronous primary malignancies of the pancreas have been reported in the medical literature. We report a case of two solid malignant tumors of the pancreas diagnosed preoperatively.

**Case presentation:**

We describe a 65-year-old Sri Lankan woman who presented with progressive obstructive jaundice. Initial contrast-enhanced computed tomography imaging detected a malignant tumor at the tail of her pancreas. A second tumor of the pancreatic head was detected with integrated imaging using multidetector computed tomography and multimodal magnetic resonance imaging. She underwent total pancreaticoduodenectomy and splenectomy. Gross examination of the specimen confirmed the presence of two separate tumors. Histology of the ampullary tumor showed pancreatic-type adenocarcinoma and the tumor in the tail of her pancreas showed a colloid-type adenocarcinoma.

**Conclusion:**

The possibility of multiple primary malignant solid tumors of different types with malignant potential has to be considered even without background pathology when managing multiple tumors in the pancreas.

## Background

The finding of synchronous primary tumors involving one organ is rare. Only a few cases of multiple solid primary pancreatic tumors have been reported in the literature [1, 2]. Most such multiple tumors were found in previously diseased pancreases with chronic pancreatitis [1]. We report our management experience of a preoperatively detected ampullary adenocarcinoma and a mucinous pancreatic adenocarcinoma involving the tail.

## Case presentation

A 65-year-old previously healthy Sri Lankan woman was referred for the management of progressive painless obstructive jaundice of 1-month duration. She had two episodes of cholangitis which had been managed with antibiotics administered intravenously. There was loss of appetite and loss of weight (12 kg/month). Her premorbid metabolic equivalent of task (MET) score was >6, but was only 3 when she was referred. She was icteric and had mild dependent edema. Her body mass index (BMI) was 17. There were no palpable abdominal masses.

**Fig. 1:**
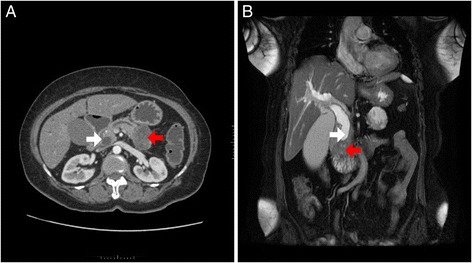
Imaging of the tumours. **a** – Axial cut of MDCT showing the two tumors of the pancreas. The ampullary tumour (17×19 mm) with ill-defined margins invading adjacent pancreatic tissue (*white arrow*) and the tumor in the tail of the pancreas (28×38 mm) with irregular margins (*red arrow*). **b** – A coronal MRI image showing the dilated CBD with smooth tapering stricture at the distal end (*white arrow*) and the ampullary tumour inferior to the distal end of CBD (*red arrow*)

She had biochemical evidence of obstructive jaundice: total bilirubin 3.4 mg/dL, direct bilirubin 3.0 mg/dL, and alkaline phosphatase (ALP) 624 IU/dL. Contrast-enhanced computed tomography (CECT) only identified a 26×37 mm tumor in the pancreatic tail with a mildly dilated common bile duct (CBD) without an apparent cause for it. Since she was unfit for major surgery an endoscopic retrograde cholangiopancreatography (ERCP) was performed, which demonstrated a stricture with smooth tapering at the distal end of her CBD. The ampulla was prominent but there was no evidence of a mass lesion from which to take a biopsy. Brush cytology from the stricture was negative for malignant cells. A 6 cm self-expandable metal stent (SEMS) was inserted. Following the endoscopic intervention, her icterus disappeared and her liver profile improved. In order to find out the cause for the distal CBD stricture, a second computed tomography (CT) scan combined with multimodal magnetic resonance imaging (MRI) of her abdomen was performed. This combined CT/MRI of her abdomen detected a second tumor in her pancreas; it was a mass with ill-defined margins of 17×19 mm, which invaded adjacent pancreatic tissue just inferior to the distal end of her CBD. Her superior mesenteric artery or vein was not involved (Fig. 1).

Her American Joint Committee on Cancer (AJCC) stage was 2A for both tumors (ampullary tumor T_3_, N_0_, M_0_ and the tumor of the tail T_2_, N_0_, M_0_). The decision to offer total pancreaticoduodenectomy was taken in a multidisciplinary team (MDT) meeting because of the anatomical location of the two tumors. She was referred to a nutritionist and was optimized with supplementary parenteral nutrition for 3 weeks. She was also vaccinated to prepare her for a possible splenectomy. Her performance status improved within 3 weeks and a total pancreaticoduodenectomy and splenectomy were performed. Her postoperative period was complicated by a lower respiratory tract infection, superficial surgical site infection, and poor glycemic control. She was managed in a surgical ward with the support of endocrinology, microbiology, and nutrition teams and was discharged on the tenth postoperative day. She was discharged on insulin and oral penicillin for prophylaxis. She lost 7 kg of weight postoperatively but her weight stabilized after the introduction of a special dietary regimen which included six to eight small frequent meals with energy-dense snacks and limitation of food rich in carbohydrates and fat.

A macroscopic examination of the resected specimen revealed two distinct tumors in her pancreas. One was an ampullary tumor measuring 30×30×28 mm. The second tumor was found at the tail and measured 50×42×40 mm. The distance between the two tumors was 40 mm.

**Fig. 2:**
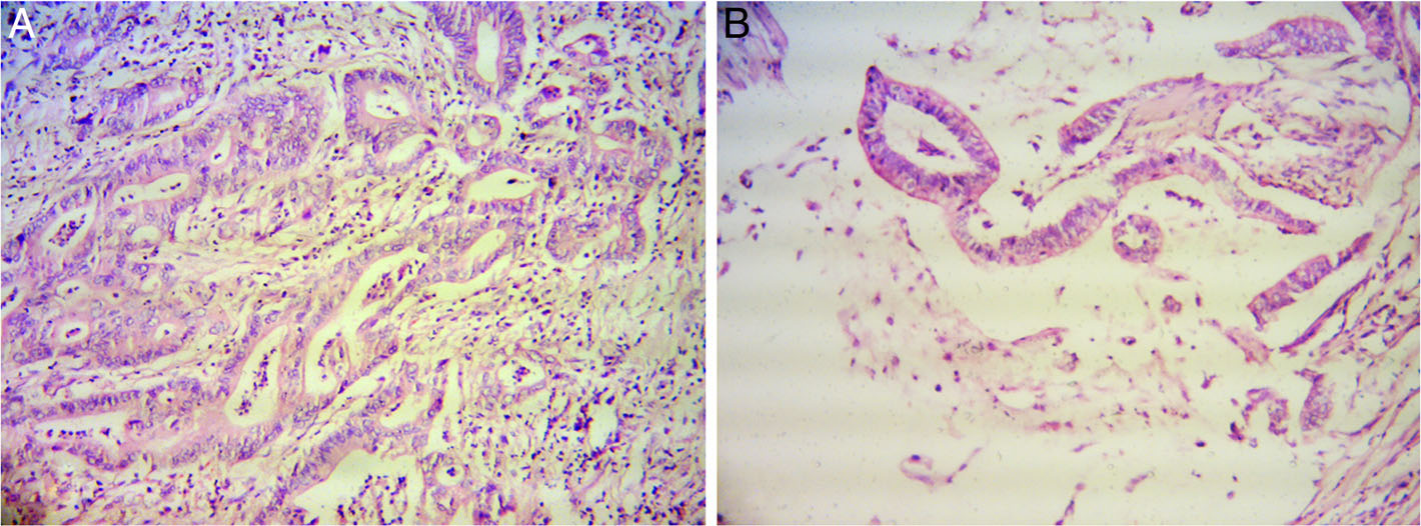
Histology of two tumors. **a** Ampullary carcinoma (hematoxylin and eosin ×200) is a moderately differentiated adenocarcinoma. **b** Hematoxylin and eosin, ×200, shows an adenocarcinoma with tumor cells suspended in pools of extracellular mucin

An histologic examination of the ampullary tumor (Fig. 2a) showed a moderately differentiated ampullary adenocarcinoma of pancreaticobiliary type. This tumor had invaded the muscularis propria of her duodenum and the pancreatic parenchyma. Her CBD was not infiltrated by the tumor. Vascular and perineural invasion were not seen. The tumor at the pancreatic tail (Fig. 2b) showed a moderately differentiated non-cystic mucinous (colloid) adenocarcinoma. Perineural invasion was seen, but vascular invasion was not detected. A few areas of chronic pancreatitis were noted in the rest of the pancreas but largely the morphology of the pancreas was normal. Immunohistochemistry findings are shown in Table 1.

**Table 1:** Immunohistochemistry of the two tumors (Dako®)

Stain	Cytokeratin 7	Cytokeratin 20	Carcinoembryonic antigen	Epithelial membrane antigen
Tumor 1 (ampulla)	Strongly positive	Negative	Focally positive	Strongly positive
Tumor 2 (tail of pancreas – colloid carcinoma)	Strongly positive	Focally positive	Strongly positive	Strongly positive

She had good quality life after surgery reaching her premorbid MET score of 6 and had a stable weight. However, her serum albumin level was persistently low (median 26 mg/dL). She died of severe pneumonia 8 months after surgery.

## Discussion

Synchronous primary tumors of the pancreas are rare and most of them were found in previously diseased pancreases with chronic pancreatitis or associated with premalignant lesions like intraductal papillary mucinous neoplasm (IPMN). An association of IPMN with multifocal ductal adenocarcinoma is well established. Yamaguchi *et al.* reported that 20% of cases in a series of 765 patients who underwent surgery for IPMN had pancreatic ductal adenocarcinoma either derived from IPMN or concomitant with IPMN [3]. Solid pseudopapillary neoplasm too is synchronously found with IPMN [4]. However, multifocal involvement in a previously healthy pancreas has not been reported to the best of our knowledge. 

A case of two separate malignant tumors of the pancreas was reported by Goong *et al.* where a tumor in the head and another in the tail of the pancreas were identified preoperatively [2]. Both lesions were finally recognized as ductal adenocarcinomas by endoscopic ultrasound-guided fine needle aspiration biopsy (EUS-FNAB), but the histologic separation of the two tumors could not be proven in this case as the patient had refused surgery. Sastry *et al.* reported a case of three synchronous tumors of the pancreas; an ampullary carcinoma with two other tumors was found incidentally in the head and in the uncinate process, during histologic preparation [1]. In this case, the ampullary tumor was of a poorly differentiated adenosquamous variety whereas the tumor in the uncinate process was a moderately differentiated adenocarcinoma and the tumor in the head of the pancreas was a benign neuroendocrine tumor (NET). Evidence of background chronic pancreatitis was well recognized. On immunohistochemical examination, pancreatic tumors do not have very specific markers. Those we had access to fitted with the morphological diagnosis; ampullary adenocarcinomas of pancreaticobiliary type are usually cytokeratin (CK) 7+/CK20– [5] and non-cystic mucinous (colloid) carcinoma CK7+/CK20+ [6]. Carcinoembryonic antigen (CEA) and epithelial membrane antigen (EMA) positivity is reported in both tumors [7].

For accurate diagnosis and staging of pancreatic neoplasms, integrated imaging is recommended. If multidetector computed tomography (MDCT) is the preferred imaging modality, multimodal MRI is recommended for integration in imaging for pancreatic tumors [8]. The cause for obstructive jaundice was not detected in the initial imaging in this case due to the low resolution of the CECT. After ERCP confirmed the presence of a lower CBD stricture, CECT combined with multimodal MRI (integrated imaging) was able to detect the tumor at the head of her pancreas in addition to the previously detected tumor at the tail.

## Conclusions

The possibility of multiple primary malignant solid tumors of different histological types has to be considered when managing multiple tumors in the pancreas. This case demonstrates the importance of utilizing optimum imaging facilities for accurate preoperative diagnosis in order to plan the most appropriate treatment option.

## Abbreviations

AJCC: American Joint Committee on Cancer
ALP: Alkaline phosphatase
BMI: Body mass index
CBD: Common bile duct
CEA: Carcinoembryonic antigen
CECT: Contrast-enhanced computed tomography
CK: Cytokeratin
CT: Computed tomography
EMA: Epithelial membrane antigen
ERCP: Endoscopic retrograde cholangiopancreatography
EUS-FNAB: Endoscopic ultrasound-guided fine needle aspiration biopsy
IPMN: Intraductal papillary mucinous neoplasm
MDCT: Multidetector computed tomography
MDT: Multidisciplinary team
MET: Metabolic equivalent of task
MRI: Magnetic resonance imaging
NET: Neuroendocrine tumor
SEMS: Self-expandable metal stent
